# Polycyclic Aromatic Hydrocarbons in Foods: Biological Effects, Legislation, Occurrence, Analytical Methods, and Strategies to Reduce Their Formation

**DOI:** 10.3390/ijms22116010

**Published:** 2021-06-02

**Authors:** Geni Rodrigues Sampaio, Glória Maria Guizellini, Simone Alves da Silva, Adriana Palma de Almeida, Ana Clara C. Pinaffi-Langley, Marcelo Macedo Rogero, Adriano Costa de Camargo, Elizabeth A. F. S. Torres

**Affiliations:** 1 Department of Nutrition, School of Public Health, University of Sao Paulo, 715 Doutor Arnaldo Ave, Sao Paulo 01246-904, Brazil; gloriaguizellini@usp.br (G.M.G.); simone.alves.silva@usp.br (S.A.d.S.); napinaffi@gmail.com (A.C.C.P.-L.); mmrogero@usp.br (M.M.R.); eatorres@usp.br (E.A.F.S.T.); 2 Organic Contaminant Core, Contaminant Centre, Adolfo Lutz Institute, 355 Doutor Arnaldo Ave, Sao Paulo 01246-000, Brazil; adriana.almeida@ial.sp.gov.br; 3 Laboratory of Antioxidants, Nutrition and Food Technology Institute, University of Chile, Santiago 7830490, Chile

**Keywords:** polycyclic aromatic hydrocarbons, benzo[a]pyrene, food quality, cooking procedures, food contamination

## Abstract

Polycyclic aromatic hydrocarbons (PAHs) are chemical compounds comprised of carbon and hydrogen molecules in a cyclic arrangement. PAHs are associated with risks to human health, especially carcinogenesis. One form of exposure to these compounds is through ingestion of contaminated food, which can occur during preparation and processing involving high temperatures (e.g., grilling, smoking, toasting, roasting, and frying) as well as through PAHs present in the soil, air, and water (i.e., environmental pollution). Differently from changes caused by microbiological characteristics and lipid oxidation, consumers cannot sensorially perceive PAH contamination in food products, thereby hindering their ability to reject these foods. Herein, the occurrence and biological effects of PAHs were comprehensively explored, as well as analytical methods to monitor their levels, legislations, and strategies to reduce their generation in food products. This review updates the current knowledge and addresses recent regulation changes concerning the widespread PAHs contamination in several types of food, often surpassing the concentration limits deemed acceptable by current legislations. Therefore, effective measures involving different food processing strategies are needed to prevent and reduce PAHs contamination, thereby decreasing human exposure and detrimental health effects. Furthermore, gaps in literature have been addressed to provide a basis for future studies.

## 1. Introduction

Polycyclic aromatic hydrocarbons (PAHs) comprise over 200 organic compounds containing two or more fused aromatic rings [1]. According to the number of aromatic rings, they can be classified as light (2–3 rings) or heavy (4–6 rings) compounds [2]. Environmental PAHs can originate from natural sources, such as forest fires and volcanic emissions, and from sources associated with human activity (i.e., artificial or anthropogenic sources), such as coal burning, vehicles exhaust emissions, engine lubricating oils, and cigarette smoke [3]. The pyrolytic process that generates PAHs involves three fundamental factors—high temperatures, reduced oxygen levels, and organic matter—resulting in incomplete combustion. This process generally yields a complex mixture of PAHs, which in turn can accumulate in the environment, affecting water, air, and soil, thereby entering the food chain [4,5].

Humans are exposed to PAHs through dietary and non-dietary sources (e.g., inhalation and skin contact). Among these, dietary sources represent the major exposure route. According to Rengarajan et al. [6], over 70% of the PAHs exposure of non-smokers is associated with food consumption. Importantly, PAHs can affect human health through various harmful effects, which are mostly related to carcinogenesis and mutagenesis in addition to immunosuppressive effects. Although not all PAHs are deemed carcinogens, they can still synergistically affect human health because of their role as free radicals and bioaccumulation leading to cellular damage. Recent epidemiological studies with humans and animals have indicated that the increasing cancer prevalence can be partly attributed to PAHs exposure [1,2,6]. Furthermore, other epidemiological studies have demonstrated that a large proportion of cancer cases may be ascribed to—at least in part—dietary factors, including dietary exposure to PAHs [7,8]. Therefore, the presence of these organic compounds in foods is concerning and requires continuous monitoring.

PAHs exist in several categories of food and drinks as a complex mixture comprising light and heavy compounds [2]. The European Commission has identified four major PAHs (PAH4) in foods: benz[a]anthracene (BaA), chrysene (Chr), benzo[b]fluoranthene (BbF), and benzo[a]pyrene (BaP) [9]. Food contamination by PAHs can originate from environmental pollution as well as during food preparation and processing. Regarding environmental contamination, most gas-phase light PAHs present in the atmosphere can be adsorbed into particulate matter and, in conjunction with light and heavy PAHs accumulated in the soil and water, can enter the food chain through their uptake by vegetation and plant materials. Furthermore, when contaminated vegetation is used to feed livestock, PAHs start to accumulate in products of animal origin and their derivatives [10]. Conversely, PAHs can be formed during food processing, such as smoking or drying—especially when the fuel is only partially combusted [11]—as well as during preparations involving high temperatures and/or open flames, such as grilling, toasting, roasting, and frying [2,10].

Industrial, natural, and domestic processes have increased the general population’s susceptibility to carcinogens exposure, including PAHs. The main chemical and physical properties of PAHs present in foods are summarized in Table 1. The most extensively studied PAH is BaP [2,6,12], particularly because of its genotoxic activity following the direct intercalation in the DNA, causing structural disruptions that lead to mutations [13]. The International Agency for Research on Cancer (IARC) classifies BaP as a Group 1 Agent, which are carcinogenic to humans, whereas BaA, BbF, and Chr are classified as Group 2B Agents, which are possibly carcinogenic to humans [12]. In addition to IARC, the Joint Food and Agriculture Organization/World Health Organization (FAO/WHO), Scientific Committee on Food (SCF), and European Food Safety Authority (EFSA) also classify and evaluate the carcinogenicity of PAHs according to their occurrence [14]. These organizations classify BaA, Chr, BbF, and BaP as mutagenic, genotoxic, and carcinogenic compounds.

This study aims to examine the available literature on PAHs to revisit the knowledge regarding their occurrence and biological effects as well as to compile the current analytical strategies to monitor PAHs levels, legislations, and strategies to reduce their formation in foods.

## 2. Biological Effects of PAHs

The absorption of PAHs is facilitated by their increased solubility in fats, and their lipophilic nature allows them to bind with the cell membrane. This binding causes structural changes, interfering with normal functions of the cell [16]. Among PAHs, BaP is the most easily solubilized by lipids. This compound may associate with lipid distribution molecules, such as chylomicrons and other lipoproteins, thereby permeating several systems responsible for lipid absorption and distribution and, consequently, promoting its bioaccumulation in tissues and organs that actively participate in these processes, such as the liver and small intestine [17,18].

More specifically, the distribution of PAHs depends on fatty acids associated with triacylglycerides, free cholesterol, and phospholipids incorporated in the structure of chylomicrons synthesized by intestinal cells and transported by lymph vessels. This is followed by distribution to tissues—such as skeletal muscle tissue, adipose tissue, and liver, through very low-density lipoprotein, low-density lipoprotein, and high-density lipoprotein—via blood capillaries [17,18,19].

Importantly, chronic human exposure to PAHs can explain the increase in the prevalence of some diseases, such as lung cancer in smokers and intestinal diseases in non-smokers [2,7,14]. In particular, foods containing a high content of lipids (e.g., beef, poultry, and fish) are excellent delivery systems for these molecules, allowing their passive absorption by the gastrointestinal tract [17,18,19].

The toxic effects of PAHs depend on the duration and mode of exposure. Short-term effects include skin and eye irritation, nausea, vomiting, and inflammation, while long-term effects are associated with skin, lung, bladder, and gastrointestinal cancers; kidney, and liver damage; and cataracts; as well as genetic mutation, cell damage, and cardiopulmonary-related mortality [10,20]. Furthermore, PAHs are metabolized through several pathways involving phase I and II enzymes, including the cytochrome P450 peroxidase and aldo-keto reductase pathways, generating metabolites such as diol-epoxides and radical cations. These metabolites can bind to proteins and DNA, forming DNA adducts that lead to biochemical disruptions and cellular damage, consequently causing carcinogenic, mutagenic, immunosuppressive, and teratogenic damage. These genetic mutations are associated with the development of fetal malformations and tumors [21,22,23,24]. In addition, considering that PAHs commonly exist as a complex mixture of different structures, they can exhibit synergistic effects, possibly increasing their toxicity [2]. Recently, new evidence showed that the intestinal microbiota modulates the activity of xenobiotics such as PAHs. The host microbiota may exacerbate the toxic activity of PAHs through pathways such as the enterohepatic cycle, as well as through changes in gene expression by liver enzymes such as P450. However, the specific mechanisms involved in this microbiota-mediated modulation remain unclear [25].

**Figure 1:**
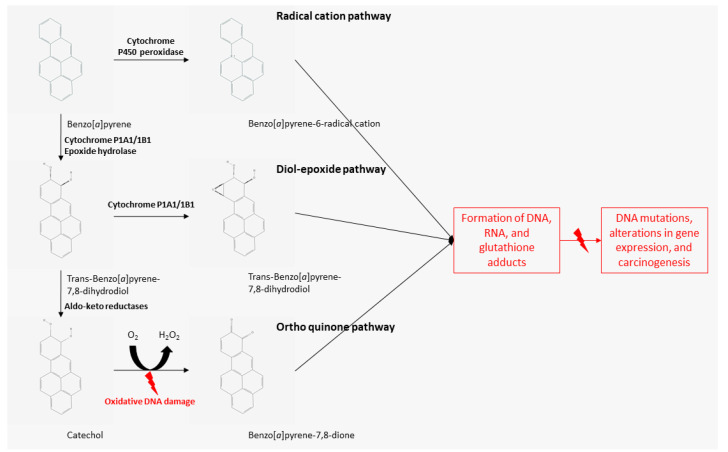
Representation of different pathways of polycyclic aromatic hydrocarbon activation and its effects on human health. Based on information from [17,19].

The metabolism and biotransformation of PAHs are complex. They are metabolized by several enzymes through different pathways. Among these, the diol-epoxide, radical cation, and ortho quinone pathways are involved in the activation of PAHs into carcinogens (Figure 1). Specifically, in the diol-epoxide pathway, phase I enzyme cytochrome P1A1/1B1 and phase II enzyme epoxide hydrolase transform BaP into dihydrodiol epoxides, i.e., (+)benzo[a]pyrene-7,8-dihydrodiol-9,10-epoxide. These newly formed “activated” PAHs have increased polarity and electrophilicity, conferring them increased reactivity. If not phagocytosed by macrophages and excreted in feces and urine, the activated products of these metabolic pathways (radical cations, diol-epoxides, and *o*-quinones) can form DNA, RNA, and glutathione adducts, thereby inducing mutations, alterations in gene expression, and carcinogenesis [17,19].

Entities such as the IARC, WHO, European SCF, United States Environmental Protection Agency (US-EPA), Agency for Toxic Substances and Disease Registry (ATSDR), and European Union have recognized the risks to human health associated with PAHs exposure. These agencies have emphasized the importance of monitoring PAHs levels according to their occurrence and toxicity to humans [14,26,27].

### Carcinogenicity of PAHs

PAHs can cause mutations in chromosomes, affect fusion and junction processes, and potentially induce chromosomal breaks. Consequently, the genetic material stored in these cells becomes unstable. If DNA repair mechanisms do not occur, the genotoxic agent will permanently affect the cell’s DNA during the initiation phase of the transcription process, generating an unpreventable mutation that will result in a pre-neoplastic cell [13,28].

The repair mechanisms triggered before the formation of a pre-neoplastic cell can restore damaged cellular DNA. DNA damage sensors—including ataxia telangiectasia mutated and ataxia telangiectasia Rad3-related—recognize changes in the genomic sequence and initiate effector repair mechanisms. Apart from initiating repair mechanisms, these kinase enzymes halt the cell cycle via the catalyzation of substrates’ intermolecular phosphorylation. They also activate the tumor suppressor gene p53, which can affect other cell regulatory genes that induce DNA repair in addition to stimulating apoptosis [29].

Failure of one or more of the suppression systems or constant and excessive stimulation by genotoxic agents can result in the full development of pre-neoplastic cells. These cells are resistant to apoptosis and, with the promotion of clonal expansion, they generate benign tumors of detectable cell mass. Subsequently, these cells acquire autonomous growth capacity for an undetermined time, which can induce angiogenesis and affect the tumor’s metastatic capacity. Consequently, a malignant mass capable of reprogramming metabolic pathways for its expansion and repelling the action of immune cells may be formed [13,28].

The IARC has assessed the carcinogenicity risks associated with exposure to complex mixtures, lifestyle factors, biological and physical agents, and specific professions since 1983 [30]. The Joint FAO/WHO Expert Committee on Food Additives classified the following 13 PAHs as genotoxic and carcinogenic: BaA, Chr, CPP, BbF, BaP, 5-MC, BjF, BkF, DBA, IP, DeP, DiP, and dibenzo[a,h]pyrene [31]. Among these, BaP was recognized as the compound with the most significant carcinogenic effect [32]. In 2010, the IARC classified BaP as a carcinogen (Group 1), while BbF, BaA, and Chr were classified as possible carcinogens (Group 2B) with limited evidence of carcinogenicity in humans [12] (Table 1).

The IARC and EFSA have identified 16 important PAHs (Table 2) that are both genotoxic and carcinogenic. Of these, they selected four PAHs (BaA, Chr, BbF, and BaP) to represent good indicators of their toxicity and occurrence in food, and eight PAHs (BaA, Chr, BbF, BkF, BaP, IP, DBA, and BPe) for risk assessment [31].

## 3. Legislation Concerning PAHs in Food

Considering the toxicity of PAHs, several countries have drafted legislation to establish tolerable limits for PAHs in foods, food products, and beverages as well as to enforce monitoring strategies for the most relevant compounds. Furthermore, health agencies such as the WHO and European Commission have launched efforts to decrease the concentration of PAHs in food, especially through strategies to control the processes that induce their formation [5,9,12,32,33].

Until 2008, the EFSA used BaP as the only marker for PAHs occurrence in food. However, a reevaluation study revealed that 30% of food samples with low levels of BaP had significant levels of other possible carcinogens [34,35]. Therefore, the European Union amended the previous legislation through Commission Regulation 835/2011, which introduced acceptable levels for the sum of PAH4 (BaA, Chr, BbF, and BaP) while maintaining a separate maximum level for BaP to ensure comparability with previous and future data [9].

The current European legislation provides specific parameters for edible fats and oils; cocoa beans and derived products; smoked meat and meat products; smoked fish and fish products; smoked bivalve mollusks; processed cereal-based foods and baby foods for infants and young children; infant formula and follow-on formula; special dietary foods; cocoa fiber and cocoa fiber products; banana chips; food supplements containing propolis, royal jelly, spirulina, or their preparations; dried herbs and dried spices except for cardamom; and smoked *Capsicum* spp. and smoke flavorings [9,36,37,38].

Concerning vegetable oils, coconut oil can contain higher levels of PAH4 than oils and fats from other sources because coconut has a proportionally higher concentration of BaA and Chr, which cannot be easily removed during coconut oil refinement processes. Accordingly, a 2008 EFSA document stipulated limits of 2.0 and 10.0 µg/kg for BaP and PAH4, respectively, in vegetable oils and fats, whereas the limit for PAH4 in coconut oil is 20.0 µg/kg [9,35].

In 2012, new directives on PAHs in traditionally smoked meats and meat products were introduced in Europe. These directives included a reduction in the acceptable levels of BaP and PAH4 in these types of food. However, certain European Union Member States could not comply with the limits on traditionally smoked meats. Therefore, in accordance with the Commission Regulation 1327/2014, these countries could continue using traditionally smoked meats and their products containing BaP and PAH4 levels higher than 5.0 and 30.0 µg/kg, respectively, as stipulated by the Commission Regulation 835/2011 [36].

In 2020, the Commission Regulation 1255/2020 reviewed the maximum levels in traditionally smoked meat and derived products as well as in traditionally smoked fish and derived products [37]. After the Commission Regulation 1327/2014 [36], the European Union Member States have monitored PAHs levels in traditionally smoked products and implemented strategies to improve smoking practices. After a new assessment conducted in 2018, the European Commission concluded that improving smoking practices was insufficient to reduce the concentrations of PAHs to permitted levels. Therefore, they granted an undetermined extension for local production and consumption of smoked products from these Member States, allowing BaP and PAH4 levels above 5.0 and 30.0 µg/kg, respectively [37]. Additionally, the new regulation established a maximum level of 10.0 and 50.0 µg/kg for BaP and PAH4, respectively, in plant-based powders used for beverage preparations [37].

In other countries, legislation concerning acceptable PAHs levels in food remains insufficient. In Brazil, legislations enacted by the Ministry of Health stipulate limits for BaP in drinking water (0.7 µg/L), smoke aroma used in foods (0.03 µg BaP/kg food), and olive pomace oil (2 µg/kg) [39,40,41]. Apart from these, Brazilian regulatory agencies have adopted the resolutions instituted by the European Commission because of the lack of regulation regarding PAHs limits for other foods and beverages [9,36,38]. In the United States, the Environmental Protection Agency (EPA) established a legal limit of 0.2 µg/L of BaP in drinking water under the National Primary Drinking Water Regulations [42]. However, the US has no regulations regarding PAHs levels in food. In Canada, Health Canada has established a list comprising regulatory limits for certain contaminants and other adulterating substances found in specific foods. These include limits of 3.0 µg/kg and 0.04 µg/L of BaP in olive pomace oils and drinking water, respectively [43].

## 4. Main Factors Influencing PAHs Contamination in Foods

Contamination of foods with PAHs may occur at different stages from farming (e.g., uptake of PAHs present in soil and water by crops) to processing and cooking (e.g., high-temperature cooking methods, and roasting and drying processing of foods) [44]. During the latter stages, PAHs generation depends on the product type and processing method, as well as factors such as combustible material, oxygen level, and smoke generator [3]. Nonetheless, as emphasized by Domingo and Nadal [45], it is essential to know the content of carcinogenic compounds in raw and unprocessed foods before assessing the influence of cooking techniques because contamination may occur during previous stages.

### 4.1. Cooking

Apart from the inherent characteristics of different food types (e.g., lipid content), certain cooking techniques contribute considerably to PAHs formation in foods. Specifically, roasting, frying, and grilling can form PAHs, especially when involving elevated temperatures [14,46]. Cooking techniques involving heating comprise two major types: dry and moist heat cooking (Table 3). Furthermore, other factors influencing PAHs levels in foods include proximity to the heat source and exposure duration [1,11,47].

Several factors determine the technique used during food preparation and processing, including those regarding nutritional, microbiological, and sensorial quality. Moreover, safety aspects concerning the formation of harmful compounds, such as cholesterol oxides and PAHs, also play a role in this determination [14,48]. The use of heat to prepare and process food provides several benefits. The sensorial quality increases owing to changes in texture, visual appeal, aroma, and flavor; the nutritional quality improves with the increase in digestibility and nutrients bio-accessibility; and the microbiological quality improves with the elimination of bacteria and other potentially harmful microorganisms.

In contrast, certain cooking processes can also facilitate the formation of injurious compounds. For example, amino acids, sugars, and creatine present in meat react at high temperatures to form aromatic heterocyclic amines, which have demonstrated mutagenic and carcinogenic potential. The Maillard reaction—which is responsible for the desirable flavor, color, and aroma of browned foods—between asparagine and reducing sugars can generate acrylamide as a by-product, a compound that the IARC classifies as carcinogenic. Finally, the non-enzymatic oxidation of susceptible lipids generates cholesterol oxides, which have atherogenic potential [12,14,49].

Rose et al. [50] evaluated the formation of 15 different PAHs (including the PAH4) in several samples of beef and salmon prepared by various cooking methods, including deep-frying, grilling with coal and wood, roasting in a natural gas oven, and roasting in an electric oven. Salmon and beef samples displayed the highest PAHs concentrations when grilled using mineral charcoal and wood, respectively. Moreover, high concentrations of BaP were evident when the cooking time exceeded 10 and 8 min for salmon and beef samples, respectively, demonstrating that these animal products are susceptible to PAHs formation and contamination. This susceptibility may be attributed to the high content of lipids in these products. Specifically, fish such as salmon, hake, and tuna have a higher lipid content than other fishes; thus, they display an increased susceptibility to PAHs formation during domestic cooking [50,51,52].

Alomirah et al. [43] evaluated the concentrations and profiles of 16 PAHs in various grilled and smoked foods. They detected eight genotoxic PAHs; among these, Chr (4.88 µg/kg) and BaA (2.27 µg/kg) had the highest mean values. According to the authors, the factors that most influenced the formation of PAHs were the heat source type, grilling duration, grill geometry, and marinating sauces used, as well as their fat content. Kazerouni et al. [53] analyzed meat products and other foods prepared by different cooking techniques under controlled conditions. Well-done steaks and hamburgers had the highest levels of BaP, whereas meats grilled at medium heat displayed the lowest levels of BaP. The authors estimated that the consumption of grilled meat contributes to approximately 21% of the average daily BaP intake.

Park et al. [54] evaluated the BaP levels in pork meat cooked with different techniques, including charcoal grilling, frying, and electric heating. According to their results, the only cooking method that generated significant BaP levels was grilling. Furthermore, they observed that meat grilled directly over the charcoal fire had a higher BaP level than that of meat grilled indirectly exposed to the heat source (using a modified charcoal grill concerning charcoal position, air circulation, and cap use).

Regarding the heat source type, bread toasted using indirect thermal sources, such as electrical resistance, displayed lower levels of contamination than those using flame. In indirect thermal sources, PAHs formation is mainly caused by macronutrients pyrolysis; in direct thermal sources (i.e., flame), in addition to pyrolysis, PAHs are also deposited from combustion smoke [46,55]. Furthermore, Min et al. [56] reported that PAHs formation in meat products submitted to heating is more affected by changes in temperature than exposure time.

### 4.2. Smoking

Smoking is used to impart specific organoleptic qualities to food as well as to aid their preservation. During this process, PAHs contamination occurs when volatile compounds generated by the combustion of different materials penetrate the food [57]. The contamination and concentration of PAHs in smoked products depend on several factors, including smoking technique, fuel type (charcoal or biomass), and smoking conditions (humidity, temperature, time, and airflow) [26].

Traditionally, direct smoking—i.e., the smoking process in which the food and wood smoke are placed in the same chamber—can be cold or hot. In cold smoking, the fire is not maintained, and temperatures can reach up to 30 °C. In hot smoking, the fire is maintained throughout the process and temperatures can reach up to 130 °C [26]. Alternatively, indirect smoking—i.e., the smoking process in which food and smoke are in different chambers—is most commonly used in the food industry. This process is considered safer, and the smoke can be produced in generators by friction or electrostatic [26]. Furthermore, smoke flavoring is often used in the food industry to improve organoleptic characteristics of nontraditionally smoked products. This smoke flavoring, or liquid smoke, is produced from condensed smoke and has lower PAHs levels compared with products directly smoked [14].

The CAC/RCP 68/2009 [58] specifies 10 variables that must be controlled to minimize and prevent the contamination of meat products with PAHs during smoking. These variables include fuel type, smoking method (direct or indirect smoking), smoke generation process (pyrolysis temperature and airflow), distance and position of the food in relation to the heat source, fat content of the food and how it changes during smoking, smoking time, temperature during smoking, equipment cleanliness and maintenance, smoking chamber design, and equipment [58]. The food industry can use this code based on Hazard Analysis Critical Control Point (HACCP) principles as a reference to reduce contamination during smoking and direct drying processes. The European community also published guidance documents, such as the European Salmon Smokers Association European Guide, which contains good practices for smoking, salting, and marinating fish products [59].

Various types of combustible materials can be used to produce smoke, such as coconut husks, corn ears, fruit stones, coal, and different wood types (pine, beech, and oak). Importantly, distinct materials generate smoke with different chemical profiles, including different levels of PAHs. For example, apple and alder shells generate lower PAHs levels than spruce wood [26]. Regarding smoking conditions, the distance and position of the product in relation to the smoke source also affect PAHs contamination. Roseiro et al. [60] observed that the concentration of PAHs decreased as the distance between food and smoke source increased.

Cured products—ham, salami, pepperoni, bacon, loin, among others—are smoked to impart characteristic flavor and aroma, as well as to extend their shelf-life. In a study by Škaljac et al. [61], sausages were submitted to direct and indirect smoking using temperature, fuel type (different wood types), humidity, and smoking time as variables. They observed the highest PAHs levels in products subjected to traditional direct smoking.

Tongo et al. [62] assessed the concentration of PAHs in smoked fishes obtained in local markets in southern Nigeria. The authors reported that the levels of PAH4 in these samples exceeded those recommended by the European Commission, indicating that the consumption of smoked fish represents a high risk for PAHs exposure. The study also showed that PAH4 levels varied according to region, with fish originated from West Nigeria displaying the highest PAHs contamination. Nonetheless, other studies indicated that smoked fish has lower levels of PAHs than smoked beef [63], smoked pork, and derived products [64]. These beef and pork products displayed PAHs levels above those deemed acceptable, thus posing a risk to consumers [63,64].

### 4.3. Other Processing

#### 4.3.1. Processing of Vegetable Oils

The lipophilic nature of PAHs facilitates the contamination of fats and oils. These products can become contaminated through several routes: atmospheric deposition of PAHs on plants, uptake of PAHs present in the soil by plants, oilseeds drying with combustion smoke, and solvent used for oil extraction [65,66].

Vegetable oils can be extracted from seeds, nuts, and fruits. The production involves three stages: preparation of the raw material, extraction of crude oil, and refinement [65,67]. In the first stage, the raw material is submitted to drying processes to control moisture levels; this represents an important source of contamination for vegetable oils. Drying processes can be natural (sun and wind) or artificial (using combustion to generate heated air). In the former, PAHs contamination originates mostly from environmental pollution, such as contaminated soil, water, and/or air. In the latter, contamination occurs upon contact with combustion products [66,68,69]. De Lima et al. [70] observed that corn grains dried using firewood as fuel have high concentrations of PAHs, which could consequently result in elevated levels of contamination in extracted vegetable oils.

#### 4.3.2. Drying and Roasting in Grains

Grains must undergo a drying process to reduce their metabolism and decrease the potential of developing microorganisms, thus preventing spoilage during storage. Artificial drying is the most efficient process for this purpose and is commonly used in the industry. Among fuel sources for artificial drying, wood represents the most widely used. In fact, approximately 50% of the wood produced worldwide is used for drying, generating approximately 1.8 billion cubic meters of air [71]. The Code of Practice of the Codex Alimentarius Commission (CAC/RCP 68/2009) [58] offers guidelines for direct drying, including the use of solar heat, wind, or other fuels during this process. According to the document, indirect drying can minimize PAHs formation, and the HACCP system may be applied to further prevent the formation of these contaminants in foods.

De Lima et al. [70] analyzed PAHs contamination in corn grain samples dried at air temperatures of 60 °C, 60 °C and 80 °C, and 80 °C. They detected fluorene, phenanthrene, anthracene, fluoranthene, pyrene, BaA, and Chr in the samples. The levels of fluorene, phenanthrene, and anthracene decreased with an increase in temperature, while those of Chr showed a slight increase. Moreover, the authors identified two types of PAHs in the control corn grain samples (not submitted to drying), demonstrating the contamination of grains prior to processing. Importantly, the levels of PAH4 surpassed those permitted for cereal-based processed foods according to European legislation. Furthermore, the authors concluded that PAHs contamination in grains dried using firewood as fuel depends on exposure time.

Bertinetti et al. [71] evaluated the effect of four heating sources—wood, rice husk, liquefied petroleum gas, and electric heating—on the contamination of rice grains with 16 PAHs. They concluded that air temperature (40 °C, 60 °C, and 80 °C) had little influence on PAHs levels. In contrast, different heating sources generated considerably different levels of total PAHs contamination: wood, 131.6 µg/kg; rice husk, 45.7 µg/kg; liquified petroleum gas, 15.9 µg/kg; and electric heating, 7.7 µg/kg.

While the drying process represents a source of PAHs contamination, the roasting process in coffee beans can reduce or at least not affect PAHs formation. Tfouni et al. [72] reported no statistically significant difference in PAHs levels of coffee brews produced from beans roasted at different temperatures. Ciecierska et al. [73] described that roasted coffee beans had significantly lower levels of contamination than green coffee beans, indicating that the roasting process can significantly reduce the levels of PAHs in the final product. This is attributed to the high volatility of light PAHs.

## 5. PAHs in Food and Their Impact on Food Quality

Table 4 summarizes the findings of recent studies concerning PAHs concentrations according to food type and processing method. The levels of PAHs in popularly consumed foods were presented, such as oils, dairy products, fruits and vegetables, meat and meat products, fish, and cocoa and chocolate.

### 5.1. Oils

Several studies have investigated the presence of PAHs in different commercial vegetable oil types [65,68,69,96,97,98,99,100]. Pandey et al. [96] evaluated 296 vegetable oil samples commercialized in India, including oils from nonconventional sources. They reported that extra-virgin olive oil and refined vegetable oils displayed the highest and lowest PAHs levels, respectively. Moreover, the authors indicated that all linseed and safflower oil samples and 90% of coconut oil samples were contaminated with PAHs. Chr was the most common hydrocarbon in linseed (20.7 µg/kg) and safflower (26.6 µg/kg) samples. BaP was found in 25.5% of samples, displaying the highest concentration in safflower oil samples (2.6 µg/kg).

Ciecierska and Obiedzinski [69] analyzed cold-pressed nonconventional vegetable oils, including linseed and safflower oils. They reported a PAHs content ranging from 23.41 to 234.30 µg/kg, with phenanthrene as the most frequent individual contaminant. Meanwhile, BaP concentrations ranged from undetectable to 15.74 µg/kg. Roszko et al. [68] analyzed linseed and borage oils and reported that PAH4 levels remained below 10 µg/kg.

Gharby et al. [98] evaluated the effect of roasting olive fruits on PAHs levels in olive oil. They detected BaA (2.19–31.3 µg/L), Chr (2.44–14.85 µg/L), BaP (0.51–1.59 µg/L), and BbF (0.26–2.92 µg/L). Although PAHs were present in olive oil produced from unroasted fruits, their levels increased significantly when the olives were subjected to roasting under elevated temperatures (approximately 130 °C).

In another study with olive oil, Kiralan et al. [100] evaluated the influence of chemical refining parameters on the removal of 15 PAHs. The authors assessed each step of the refining process (degumming, neutralization, bleaching, and deodorization). They deemed degumming (the step in which phosphatides, or gums, are removed from crude oil) as a crucial step in the refining process and tested different concentrations of phosphoric acid and water during this process. They reported that PAHs levels decreased 82% when using a solution containing 1% (*v*/*w*) of water. Similarly, they observed that the total PAHs content decreased 90% after the neutralization process. Furthermore, increasing the amount of activated carbon from 0.3% to 0.9% in the bleaching step was also effective in reducing PAHs levels (from 86% to 91%). Finally, the deodorization process mainly removed light PAHs and increasing the temperature during this process was ineffective in decreasing total PAHs levels. These findings are important for the development of new processing strategies for refining high-quality olive pomace oil.

Silva et al. [99] investigated PAH4 contamination in nonconventional vegetable oils (coconut, safflower, evening primrose, and linseed oils) commercialized in Sao Paulo, Brazil. They identified Chr as the most frequent hydrocarbon in these samples. Furthermore, PAH4 were detected in 96% of samples, with levels ranging from undetectable to 14.99 μg/kg. Considering the permitted limits established by European Regulation 835/2011 [9], 12% and 28% of samples had unsatisfactory levels of BaP and PAH4, respectively, and the authors attributed these unacceptable results to samples of adulterated oils.

### 5.2. Meats and Meat Products

Martorell et al. [101] analyzed the levels of PAHs in several foods consumed by the population of Catalonia, Spain, to estimate their exposure to these compounds. They observed that the consumption of meat and meat products was responsible for almost 50% of the total estimated PAH intake, with an average of 38.99 µg/kg. Similarly, Yu et al. [102] analyzed the levels of PAHs in foods available in Beijing, China, and the consumption of meat (beef, pork, poultry, and lamb) was deemed the main contributor to PAHs intake (34.4 µg/kg).

Several studies [60,63,103,104,105] reported high levels of PAHs in meats and derived products. The levels of BaP in beef [104] and ham, bacon, and fish [63] reportedly exceed those specified by European Regulation 835/2011 [9]. Furthermore, BaA and Chr were the most frequent PAHs and had the highest concentrations among the PAH4 identified [63,104]. Wretling et al. [63] also reported that ham (58.4 µg/kg) and bacon (48.9 µg/kg) exceeded the PAH4 levels considered acceptable.

Wongmaneeratip and Vangnai [47] investigated the formation of PAHs in grilled chicken breast using different marinades during charcoal grilling. After grilling each side of the breast samples for 3 min, PAHs levels increased from 190.1 to 457.6 μg/kg. The use of an alkaline marinade (pH > 7.5) increased the formation of heavy PAHs and BaP by over 70% and 80%, respectively. Moreover, adding oils to the marinade increased heavy PAHs formation, especially BbF. The authors also observed slight differences in PAHs levels when the chicken breasts were prepared using sunflower- or palm-oil marinades. They concluded that the presence of natural and synthetic antioxidants in sunflower oil is related to the reduced formation of PAHs when using this product.

Lu et al. [106] examined the formation of PAHs in chicken and beef meatballs prepared using a spice mix (0.5% garlic, onion, red chili, paprika, ginger, and black pepper powders) and deep-fried at 180 °C. They observed that the addition of spices affected the formation of BaA and BaP, as well as the total PAHs content, in both samples. The type of meat significantly influenced the formation of BaA, which was only inhibited by ginger powder. Thus, the authors concluded that the addition of spices to processed meat products could reduce PAHs formation. Furthermore, they determined that the antioxidant capacity of each spice was a key indicator of their inhibitory efficiency.

Yao et al. [107] analyzed the levels of BaP in grilled chicken drumsticks using electric and charcoal grilling. Prior to cooking, the samples were marinated with green and white tea. The authors observed that, in general, the grilling methods and tea marinades significantly affected the formation of BaP. Thus, they suggested the use of tea marinades as a pretreatment flavoring to decrease the generation the BaP during cooking.

Tongo et al. [62] evaluated the concentration of PAHs in smoked fish (*Clariasgariepinus*, *Ethmalosa fimbriata*, *Tilapia zilli*, and *Scomberscombrus*) obtained in markets in southern Nigeria. They observed that different fish species had different total PAHs levels: *C. gariepinus*, 0.715 mg/kg; *T. zilli*, 0.951 mg/kg; *E. fimbriata*, 0.694 mg/kg, and *S. scombrus*, 3.585 mg/kg. Zachara, Gałkowska and Juszczak [57] analyzed canned and smoked fish, sausages, pork ham, and chicken fillets that had been traditionally and industrially smoked. As expected, they observed that traditionally smoked sausages had the highest average concentration of PAH4 (24.27 μg/kg). Finally, Roseiro et al. [60] investigated the influence of different smoking processes on PAHs contamination in chorizo; similarly, they observed that industrially smoked products had a lower level of total PAHs than those traditionally smoked (1703 vs. 3237 µg/kg).

### 5.3. Cocoa and Chocolates

Sadowska-Rociek et al. [88] examined the PAHs content in white (*n* = 6), milk (*n* = 7) and dark (*n* = 7) chocolate samples. They observed that 10% of samples displayed PAH4 levels that exceeded those stipulated by European Regulation 835/2011 [9]. Samples were mostly contaminated with light PAHs, with phenanthrene representing the most frequent compound (13.5–96.6 μg/kg). Furthermore, white chocolate samples had significantly lower total PAHs levels than dark chocolate (68.8 vs. 113 μg/kg).

Kumari et al. [90] investigated the levels of PAHs in chocolate candy. Sixteen PAHs were detected at concentrations ranging from 2.70 to 235.91 ng/g (mean, 67.62 ng/g). BaP concentrations varied from 0.35 to 12.76 ng/g (mean, 1.62 ng/g). Overall, 8% of candy samples showed BaP concentrations exceeding 5 μg/kg stipulated by European Union regulation for products containing cocoa butter [9].

Roasting is an important process in cocoa manufacturing, significantly decreasing the astringent, bitter, and sour attributes of cocoa beans. Conversely, this process is also involved in the formation of harmful compounds, such as PAHs. Agus et al. [108] indicated that roasting parameters (roasting the whole cocoa bean or its nibs at different temperatures and times) significantly affected the formation of PAH4 in cocoa. They observed that PAH4 levels increased from 0.19 to 7.73 ng/g after increasing the temperature by 80 °C and roasting duration by 40 min. These findings indicated that controlling the roasting of cocoa beans is essential to minimize the formation of PAHs during processing.

Ciecierska [109] examined the levels of 19 PAHs in several varieties of cocoa beans (Forastero, Trinitario, Criollo, and Nacional) from different countries as well as in their processed products. They observed the highest levels of PAHs in roasted cocoa beans, cocoa mass, and cocoa butter (16.69–74.15 μg/kg of fat). Moreover, the samples were predominantly contaminated with light PAHs. All raw cocoa beans displayed a relatively low level of contamination with heavy PAHs, with combined levels considerably lower than that permissible by the European Commission Regulation 835/2011 [9]. The authors concluded that the origin of these beans had low levels of environmental pollution and that sun drying was safely performed concerning PAHs contamination.

### 5.4. Fruits and Vegetables

Jia et al. [110] evaluated the levels of PAHs in vegetable samples obtained near the industrial area of Shanghai, China. They identified 16 PAHs in these samples, with concentrations ranging from 65.7 to 458.0 ng/g in leafy vegetables (romaine lettuce, Chinese cabbage, and Shanghai green cabbage), stem vegetables (lettuce), seed and pod vegetables (broad bean), and rhizome vegetables (daikon). The authors highlighted the necessity of avoiding the cultivation of vegetables, especially leafy ones, in heavily polluted areas.

Similarly, Mohammed et al. [111] evaluated the levels of 16 PAHs in leafy vegetables cultivated within the Accra Metropolitan Assembly city center. They detected the presence of acenaphthylene, acenaphthene, BaA, BaF, and BaP in all samples except garden egg leaves and Chinese cabbage, in which they detected Chr and pyrene, respectively. Conversely, naphthalene was detected in all vegetables and the total PAHs level ranged from 0.037 to 16.2 μg/kg.

Lee et al. [15] investigated the presence of eight PAHs in 16 fruit and 26 vegetable samples obtained in Seoul, Korea. While they did not detect BaP in fruits, the compound was detected in vegetables (0.05 µg/kg). The total PAHs levels were 0.67 and 0.82 µg/kg in fruits and vegetables, respectively.

### 5.5. Milk, Dairy, and Baby Foods

Several studies investigated the presence of PAHs in milk, dairy, and baby [74,77,80,112,113,114,115]. Fresh milk contamination is associated with environmental pollution, especially that produced by biomass burning. Milk cows are exposed to PAHs present in the water, soil, and air of dairy farms, as well as in animal feed. Consequently, the milk produced by these animals may be contaminated with PAHs [114].

Shariatifar et al. [115] evaluated the PAHs in milk and milk powder samples obtained in Iran. They observed that powdered milk had the highest mean level of 16 PAHs (2.28 ± 0.39 μg/kg), and some samples exceeded the limit stipulated by the European Commission [9]. Meanwhile, pasteurized milk displayed the lowest PAHs levels (0.87 ± 0.18 µg/kg). Importantly, the authors noted a clear seasonal influence on the level of these compounds, with samples collected in winter displaying the highest levels of PAHs. This finding highlights the effect of biomass burning pollution in milk contamination.

Sun et al. [114] investigated the concentrations of PAHs in milk samples from nine countries. They determined that milk samples from Europe and Oceania displayed concentrations of 16 PAHs and BaP varying between 7.34–13.60 µg/kg and 0.46–0.83 µg/kg, respectively. All European milk samples had PAH4 exceeding the limits stipulated by the European Union, but BaP levels were below (PAH4, 2 µg/kg; BaP, 1 µg/kg) [9]. Furthermore, the concentrations of 16 PAHs in milk samples from France and Slovenia were considerably higher than those in milk samples from other countries, with no differences between organic and non-organic varieties.

PAHs contamination in milk is also affected by different sterilization methods (ultra-high-temperature processing [UHT] and pasteurization) and skimming levels. According to Sun et al. [114], skimming is effective in removing PAHs with five-to-six aromatic rings. Naccari et al. [80] reported that the levels of PAHs in raw milk were lower than those in pasteurized milk (5.428 vs. 6.519 ng/g of milk). Furthermore, they observed that whole UHT milk samples contained higher PAHs levels than semi-skimmed UHT milk (7.753 vs. 5.941 ng/g of milk).

Santonicola et al. [74] evaluated 22 milk-based baby foods obtained in Italy. In total, they observed that the samples had an average 14 PAHs concentration of 52.25 µg/kg. Importantly, 18.2% and 77.7% of samples had levels of BaP and PAH4, respectively, that exceeded those considered acceptable by the European Union [9]. These findings highlight a concerning health risk associated with the consumption of milk-based baby foods. Similarly, Badibostan et al. [112] analyzed infant formulas, follow-on formulas, and baby foods obtained from Iranian markets. Although they did not detect BaP, one of the samples exceeded the limit of 1 µg/kg stipulated by the European Commission Regulation for PAH4 (1.43 µg/kg) [9].

According to Pluta-Kubica et al. [113], the PAHs contamination of smoked cheese depends on the material used during the smoking process. When investigating the contamination of traditionally smoked cheeses, these authors observed that naphthalene was the most frequent compound in almost all samples. They also indicated that small cheeses (i.e., those with a smaller cheese-to-rind ratio) displayed the highest levels of PAHs. Fasano et al. [77] observed similar findings when evaluating traditionally smoked cheese samples obtained in Spain. According to the findings of these studies, almost all PAHs were present in the cheese rind.

### 5.6. Bread and Cereals

Although studies on cereals and bread are limited, in general, these feedstocks show lower PAH4 contamination levels than other food groups. Kacmaz et al. [93] evaluated the presence of PAHs in ten samples of commercial bread and breakfast cereal. They observed that BaP and PAH4s levels were below the limits specified by the European legislation for processed cereal-based foods for infants (1 μg/kg) [9], and the concentrations of BaP and PAH4 ranged between 0.09–0.30 µg/kg and 0.11–0.87 µg/kg, respectively. In 35 samples of cereal and bread obtained in Latvia, Rozentale et al. [116] obtained BaP and PAH4 concentrations varying between 0.01–0.24 μg/kg and 0.22–1.62 μg/kg, respectively, with 14% of samples exceeding the limits stipulated by the European Union [9].

Rascón et al. [95] evaluated 16 PAHs in 22 cereal-based foodstuffs, including cookies, flour, pasta, breakfast cereal, rice, and bread. Naphthalene and anthracene were the most frequent hydrocarbons, with concentrations ranging between 13–5500 ng/kg. The levels of PAH4 in three cereal (granola, chocolate granola, and milk-filled cereal) and flatbread samples exceeded the limit stipulated by the European Commission [9]. The authors deemed that the presence of PAHs was mainly associated with the drying process applied for commercialization. They also examined the effect of home toasting bread on PAHs levels and observed that this process considerably increased contamination, especially for PAH4, which exceeded the acceptable limit.

Traditional (lavash and taftoon) and industrial bread (baguette) from different bakeries and markets in Iran were studied by Kamalabadi et al. [94]. The mean concentrations of PAH4 and 16 PAHs were 0.93 and 84.17 µg/kg, respectively, for 169 samples, and factors like baking time, temperature, and bread thickness were the major variables influencing these concentrations. Overall, the PAH4 in 3.8% and 6% of industrial and traditional bread samples, respectively, exceeded the limits of the European Union [9].

### 5.7. Other Products

Ozoani et al. [117] investigated the contamination of honey obtained in Enugu state, Nigeria. They observed BaP and PAH4 concentrations varying between 0.1–0.524 and 0.0439–3.22 mg/kg, respectively, and the determination of BaP_eq_ levels and their exposure margin indicated that the consumption of honey did not pose a significant health risk for that population.

Alcoholic and non-alcoholic beverages, including beer, distilled drinks (vodka, whiskey, rum, and gin), soft drinks, tea, coffee, wine, and juices were evaluated by Rascón et al. [118] for the presence of 16 PAHs. While beer samples contained naphthalene (340–1500 ng/L) and anthracene (320–2200 ng/L), only naphthalene was identified in wine samples (75–3600 ng/L). Meanwhile, juice samples contained naphthalene (0.29–2.6 ng/L) as well as anthracene and fluorene (3.7–1800 ng/L). One sample of apple juice and another of orange juice contained Chr (19 ng/L) and BaP (38 ng/L), respectively. Moreover, the concentrations of PAH4 did not exceed the tolerated limit of 1 µg/kg.

Another alcoholic beverage studied was cachaça, a Brazilian sugar cane spirit. Will et al. [119] measured 15 PAHs in six samples obtained in local supermarkets, detecting levels ranging from 0.65 to 12.2 μg/L, with naphthalene as the most frequent compound (detected in 83.3% of samples). Meanwhile, BaA, Chr, and BaP were not detected in these samples.

Tfouni et al. [120] determined the levels of PAH4 in 10 different types of tea obtained from a Brazilian market, including black, green, white (*Camellia sinensis*), boldo (*Peumusboldus Molina*), chamomile (*Matricariarecutita*), lemongrass (*Cymbopogon citratus*), mate (*Ilex paraguariensis*), peppermint (*Mentha piperita* L.), strawberry (*Fragaria* spp. and *Pyrus malus* L. *fruits*, and *Hibiscus sabdariffa* L. *flowers*), and flowers and fruits (*H. sabdariffa* L. *flowers*, *Pyrus malus* L. *fruits; Rosa canina* L. *flowers*, and fruits; *Ribes nigrum fruits*, *Cichorium intybus* L. *fruits*; and *Vaccinium myrtillus* L. *fruits*) teas. Among these, mate tea displayed the highest PAH4 levels (194–1795 µg/kg), followed by black (1.8–186 µg/kg), white (24–119 µg/kg) and green (3.1–92 µg/kg) teas. Moreover, 67% of samples had BaP. Meanwhile, strawberry, lemongrass, peppermint, and boldo teas had the lowest levels of PAH4. The authors observed only trace levels of PAHs in tea infusions. This finding reflected the lipophilic nature of these compounds, which limits their solubility in water.

In Poland, 68 brands of dried tea and infusions were evaluated by Ciemniak et al. [121]. They observed 16 PAHs concentrations varying between 41.5–2910.2 and 52.9–2226.0 µg/kg in dried teas and infusions, respectively. Moreover, *Camellia sinensis* teas were more contaminated than herbal and fruit teas. The mean solubilization rate of PAHs from dried teas to water ranged from 2.4 to 25.3%. The authors indicated that BaP levels in two types of black tea infusions exceeded the limit specified for water-based beverages (10 ng/L).

Rozentale et al. [116] investigated the PAH4 in dried herbs and spices (oregano, basil, thyme, black pepper, paprika, and nutmeg) samples (*n* = 150) obtained from Turkey, Brazil, China, Poland, Indonesia, and Vietnam. Most samples (86%) contained at least one hydrocarbon, with Chr the most frequent contaminant. Overall, the authors observed PAH4 and BaP levels varying from undetectable to 37.39 μg/kg and to 6.60 μg/kg, respectively. Importantly, none of the samples had PAH4 levels that exceeded the acceptable limit stipulated by the European Union [9].

## 6. Analytical Methods for the Quantification of PAHs

The methods for extraction, identification, and quantification of PAHs in food remain time-consuming and challenging. The Association of Official Analytical Chemistry Official Methodology 973.30 [122] is indicated for the analysis of PAHs in food. This methodology includes compound separation using thin layer chromatography and subsequent determination using ultraviolet spectrophotometry. However, these methods require high volumes of solvents and have low sensitivity. In the last decade, the interest in the presence of PAHs in food and its impact on human health has increased, consequently stimulating the development of more efficient procedures for their extraction and quantification [123]. Table 5 summarizes the main analytical methods used for determining PAHs in food.

In relation to other compounds present in food, PAHs exist in very low concentrations. In addition, food matrices add another layer of complexity for the analysis of these compounds. Therefore, extensive methods with several stages of sample preparation are required for analyzing PAHs [124]. Furthermore, all procedures must carefully avoid any cross-contamination. Recommended measures include attention to sampling and sample preparation, use of pure solvents and inert materials like glass, decontamination of the glassware prior to analysis, avoidance of contact with plastic material (which can adsorb PAHs or release interferents), extraction and manipulation of samples with minimal light exposure, and avoidance of high temperatures [9,125,126].

Several factors can affect the quantification of PAHs, such as pH, temperature, ionic strength, solubility in its matrix of origin, among others. PAHs are nonpolar compounds; thus, they are easily accumulated in matrices with high lipid content or other nonpolar components [127]. Accordingly, each food matrix has its specific sample preparation according to its composition. Thus, in-depth knowledge of the matrix of interest is essential for determining the appropriate steps for PAHs analysis [126].

In food products consisting mostly of fats and proteins, saponification, liquid-liquid extraction, and purification steps are necessary to isolate and quantify the PAHs [89,90,92]. For example, a saponification step is necessary when analyzing the PAHs content of meat products. During this step, the reaction with potassium or sodium hydroxide breaks down the protein and lipids present in meat matrices, thereby releasing the PAHs adsorbed on them. Subsequently, the analytes of interest are isolated using liquid-liquid extraction with organic solvents such as hexane or cyclohexane, simultaneously removing impurities and other polar compounds [14,128].

The clean-up/purification step is indispensable to isolate the analytes of interest and to remove potential interfering compounds such as triglycerides and fatty acids from the matrix. This step can be performed using solid-phase extraction techniques with organic solvents. The most appropriate sorbents are silica, C18, florisil, or a mixture of any two of these. This technique has been widely used owing to its sensitivity and speed [21,25].

Recently, methodologies using fewer and less toxic solvents have been adopted. One of these is the “quick, easy, cheap, effective, rugged, and safe” method, named QuEChERS, that is employed during the extraction step and reduces sample preparation time, analysis cost, and chemical residue generation. This method was used to analyze PAHs in matrices such as drinking water, tea, seafood, and meat products [138]. In 2014, the Association of Official Analytical Chemistry published a methodology for the determination of 16 PAHs in fish, mollusk, and shrimp using the QuEChERS principles [133]. Furthermore, several studies have used this method in the clean-up step along with other reagents such as primary secondary amine, octadecyl, and silica to remove food matrices [120,131,139,140].

More recently, Slámová et al. [135] proposed the use of an “enhanced matrix removal” method to eliminate lipids in smoked products of animal origin. According to the authors, the principle of this method is the entrapment of long-chain hydrocarbons associated with lipids within the “enhanced matrix removal” structure after the sorbent is dissolved to saturation in the sample extract solution. Using this method, they reported recovery rates between 50%–120% for all compounds with relative standard deviation values lower than 16.7%.

Regarding quantification, PAHs can be quantified using gas chromatography coupled with mass spectrometry (single quadrupole, triple quadrupole, and quadrupole time-of-flight) [15,50,56,61,63,114,120,131,132,139,140,141,142]. Albeit not as common as other methods, gas chromatography with flame ionization detector may also be used [104]. Mass spectrometry is a preferable mode of detection as it confirms the identity of the analyte with high selectivity and for using stable isotope labeled PAHs as internal standards, thereby reducing analytical errors [116].

Some PAHs naturally emit fluorescence. Exploring this characteristic, several studies have quantified PAHs using liquid chromatography with fluorescence detector [47,60,74,97,106,129,130,143,144]. Furthermore, liquid chromatography coupled with mass spectrometry is a promising technique, with studies using triple quadrupole, and Orbitrap, as well as ionization methods compatible with nonpolar compounds such as atmospheric pressure ionization photoionization [116,145]. In this method, a vacuum ultraviolet lamp emits photons and a dopant such as toluene or anisole is used to further enhance the analytes ionization. Hollosi and Wenzl [133] used this technique to analyze vegetable oils, obtaining a limit of detection of 6.3 pg of BaP on column for the certified blank oil (corresponding to 0.24 μg/kg of BaP). Additionally, Rozentale et al. [116] used the same technique to analyze dark chocolate, observing a limit of detection varying between 0.8–1.2 pg injected on column for PAH4.

## 7. Risk Assessment of PAHs in Foods

Risk assessment identifies and quantifies the hazard, exposure, and risk according to factors such as chemical agent potential toxicity, conditions, and exposure intensity. This assessment employs hazard identification, hazard characterization, exposure assessment, and risk characterization to analyze risk–benefit relationships and establish safe concentrations, supporting health surveillance strategies put forth by regulatory agencies, industries, and other organizations [146].

The risk characterization stage combines data on hazard and exposure assessments. For PAHs in food, these can be conducted using the margin-of-exposure (MOE) approach suggested by the SCF, toxic equivalency factors, and/or incremental lifetime cancer risk (ILCR) [85,147]. These assessments require dates of occurrence and data on consumption, and the overall quality of these data are paramount for the accuracy of the risk assessment.

Recently, many studies reporting data on PAHs occurrence and health risk assessment have been published investigating whether a potential risk exists when consuming certain foods. Bogdanovic et al. [85] investigated 180 samples of fish and meat products obtained in Croatia and, although they observed high levels of PAHs, they concluded that these products do not present health risks to consumers based on the MOE results.

In a study on edible vegetable oils conducted in Iran, the MOE attributed to oil ingestion varied between 2.17–4.10 × 10^5^ in adults and 2.86–5.38 × 10^4^ in children. In addition, the ILCR ranged from 4.17 × 10^−6^ to 5.20 × 10^−6^ in adults and 3.17 × 10^−5^ to 3.94 × 10^−5^ in children. Sunflower, corn, blended, and frying oils had the highest to lowest MOE associated with their consumption. Conversely, the consumption of frying, blended, sunflower, and corn oils had the highest to lowest associated ILCR. Ultimately, the authors concluded that, according to the health risk assessment, adults and children were not at a considerable health risk owing to oil ingestion (MOE ≥ 1 × 10^4^ and ILCR < 1 × 10^−4^) [148].

Another study analyzing PAHs in commercial tea and coffee samples marketed in Iran reported that the ILCR was lower than 1 × 10^−4^ (ILCR less than 10^−6^ indicates an acceptable level of risk, while a value between 10^−6^ and 10^−4^ indicates a potential health risk), indicating no significant risk of cancer attributable to the consumption of tea and coffee in children and adults [149]. Jia et al. [110] studied the daily ingestion of PAHs related to the dietary consumption of vegetables planted near industrial areas of Shanghai, China. In ascending order, the vegetables that most contributed to PAHs ingestion were Chinese cabbage, broad bean, romaine, Shanghai green cabbage, lettuce, and daikon. Moreover, the authors observed that children had higher daily PAHs intake than teenagers and adults. The ILCR of individuals that consumed those six vegetables ranged from 4.47 × 10^−7^ to 6.39 × 10^−5^ according to age and sex, indicating a potential cancer risk. These findings are based on the U.S. EPA standard, in which a one-in-a-million incremental risk of human cancer over a 70-year lifetime period (ILCR = 1.0 × 10^−6^) is the level of risk considered acceptable or inconsequential, whereas an incremental lifetime cancer risk of one in ten thousand or greater (ILCR = 1.0 × 10^−4^) is considered serious and requires close surveillance [110].

According to the MOE of PAHs, the consumption of fried and grilled fish from Shandong, China, did not represent a significant toxicological concern for consumers. The MOE for BaP, PAH4, and PAH8 are greater than 1 × 10^4^, indicating minimal health risks. However, the ILCR associated with the consumption of fried and grilled fish were greater than 1 × 10^−6^, indicating a potential health risk for the adult population [147].

A study on PAHs in human milk and infant formula conducted in Italy indicated that the MOE for BaP and PAH4 were lower than 1 × 10^4^ in 63% and 67% of breast milk samples and in 47% and 67% of infant formula samples, respectively, indicating a potential health risk [74]. Sirot et al. [150] evaluated the exposure to PAHs of non-breastfed French children under 3 years of age using data obtained from a 3-day food diary. The MOE varied between 5.7 × 10^4^ and 3.0 × 10^5^ and, considering the benchmark dose level associated with a 10% incremental risk of adverse events of 0.34 mg∙kg∙bw^−1^∙d^−1^, the authors deemed the dietary exposure of infants to PAHs tolerable. The products that contributed the most to this exposure were infant formula for children up to 12 months old and ready-to-eat baby foods with meat or fish for children over 12 months old.

## 8. Strategies to Reduce Food Contamination with PAHs

### 8.1. Marinades

Marinating meat in a mixture of condiments before cooking is a common practice to improve its flavor, aroma, and/or texture [129]. This pretreatment can influence the formation of PAHs during cooking according to the type of ingredients used: while some compounds present in these condiments can inhibit the formation of carcinogenic compounds, others can accelerate this process instead [46]. Ingredients that have antioxidant properties, such as spices, garlic, and onion, can inhibit the formation of PAHs in fried pork [46]. Adding onion (30 g/100 g of meat) or garlic (15 g/100 g of meat) to meat preparations resulted in a 60% and 54% decrease, respectively, in six PAHs levels [151].

Conversely, adding oil or alkaline ingredients to grilled chicken considerably increased PAHs levels [47]. Meat marinated for 1 h with oil (palm or sunflower) or alkaline ingredients (sodium bicarbonate, pH > 7.5) displayed an increase of over 70% and 80% in 16 PAHs and BaP levels, respectively. Importantly, the use of oil increased the levels of heavy PAHs, especially BbF (146.5 μg/kg) and BaP (84.6 μg/kg). In contrast, the addition of acidic ingredients in marinades reduced the formation of PAHs. Farhadian et al. [129] reported that treatment with oil containing lemon juice or tamarind decreased PAHs formation. The authors also observed that the pretreatment duration (4–12 h) did not influence the formation of these compounds, with 4 h already considered adequate for PAHs formation mitigation.

Wang et al. [152] analyzed the effects of six brands of beer on the formation of PAHs in charcoal-grilled chicken wings. The authors evaluated the changes in eight PAHs to determine which of these compounds were most affected by the marinade pretreatment compared with non-marinated samples. In addition, they used different marinades with distinct phenolic compositions to identify which beer-derived phenolic compounds can effectively inhibit PAHs generation. Some brands of beer displayed stronger inhibitory effects on the formation of all PAHs, which the authors attributed to higher levels of phenolic compounds and increased free radical scavenging capacity. These findings support the use of foods or edible materials rich in phenolic compounds as potential natural inhibitors of PAHs formation during cooking processes.

Another study by Wang et al. [84] identified eight phenolic compounds with positive effects on the inhibition of PAHs formation in charcoal-grilled chicken wings: epigallocatechin gallate, gallocatechin, catechin, epicatechin gallate, catechin gallate, eriodyctiol, naringenin, and quinic acid. Different marinades resulted in distinct PAHs profiles, and the PAHs concentration in the control chicken samples was twice as high as that in the samples marinated with quinic acid, the compound that exhibited the strongest inhibitory effect on PAHs formation.

El Badry [153] also evaluated the relationship between different pretreatments and the formation of PAHs in chicken. Pretreatment with a marinade comprising tomato juice, garlic paste, onion, salt and spices (cumin, coriander, and black pepper) contributed the most to PAHs reduction, followed by a pretreatment with garlic and spices (cumin, coriander, black pepper, and rosemary). Moreover, pretreatment with spices resulted in a greater reduction in PAHs formation that pretreatment with garlic alone. According to the authors, this finding may be attributed to the synergistic effect of the antioxidant compounds present in the spice mixture.

### 8.2. Cooking Methods

Bansal et al. [66] showed that alternative cooking methods can reduce PAHs formation in prepared foods. These methods included cooking at lower temperatures, exposing the lean portion of the meat during grilling, avoiding direct contact of the food with the flames during barbequing, and using electric or gas broilers instead of charcoal.

Regarding the heat source, methods that use indirect thermal sources, such as electrical resistance, have resulted in lower PAHs contamination rates than those that use flames in roasted bread samples. When using indirect thermal sources, PAHs are formed mostly through the pyrolysis of macronutrients; when using open flames, PAHs from combustion smoke are also deposited in the food [46,55]. Accordingly, Alomirah et al. [154] reported that meat roasted in an electric oven had lower levels of PAHs than meat roasted over coal.

### 8.3. Solvents Used during Extraction Processes

Galeotti et al. [155] evaluated strategies to eliminate or reduce PAHs in highly contaminated raw propolis while preserving its polyphenol compounds, the main active components of propolis. The authors observed that raw propolis was highly contaminated with eight PAHs (1260 μg/kg), including BaP (220 μg/kg). They tested eight different extracts obtained using solvents with different proportions of water and ethanol (from 100% to 30% ethanol solutions). A progressive reduction in ethanol content resulted in a considerable decrease in total polyphenol content and antioxidant activity, especially for ethanol concentrations lower than 50%. In contrast, extracts obtained with 80–100% ethanol solutions still contained levels of PAHs that exceeded the limits put forth by EFSA. Meanwhile, propolis extracts obtained with 60%–70% ethanol solutions showed good characteristics. Therefore, determining the most appropriate solvent combinations for extraction processes can represent an effective way of producing quality products with lower contamination levels.

### 8.4. Washing

Mahugija and Njale [156] investigated the effect of washing smoked fish on their PAHs levels. They observed that PAHs levels decreased by 86.5%, 56.1%, and 31.5% in washed samples of *S. victoriae*, *L. niloticus*, and *Haplochromis* spp., respectively, compared with those of unwashed samples. Depending on the fish species, washing with warm water significantly reduced or eliminated most PAHs. Therefore, the authors recommended that smoked fish should be thoroughly washed before cooking to reduce the concentrations of PAHs, thereby making the products safer for consumption. Nonetheless, the overall PAHs concentrations remained above acceptable levels even after washing.

### 8.5. Irradiation

Khalil and Mahfouz [157] investigated the effects of gamma irradiation at 0, 5, 10 and 15 kGy on 16 PAHs present in pea seeds. They observed that PAHs concentrations decreased as the gamma irradiation dose increased. The 15-kGy irradiation reduced the total PAHs levels in pea seeds by approximately 96% compared with those in control samples. Importantly, gamma irradiation did not affect the chemical composition of the samples.

### 8.6. Reduction of Smoke Contamination Used in the Smoking Process

Different types of wood and coal produce different levels of PAHs, especially of BaP. Therefore, choosing the combustible material used in traditional smoking methods is important to reduce PAHs contamination [26,60]. Moreover, the distance and position of the product in relation to the smoking source also influences this contamination.

Babić et al. [158] evaluated the impact of zeolite, granular activated carbon, and gravel filters on PAHs levels in traditionally smoked carp meat. They observed that the smoking process using zeolite filters resulted in the lowest levels of PAHs (52.42 µg/kg).

### 8.7. Food Packaging

Kuźmicz and Ciemniak [159] evaluated the influence of different packaging materials on the absorption of 16 PAHs from smoked sprats (*Sprattus sprattus*). The material with the greatest absorption was high-density polyethylene, followed by low-density polyethylene, polypropylene, oxo-degradable, and polyethylene terephthalate. When the high-density polyethylene packaging was analyzed, the authors observed a six-fold increase in the total PAHs level compared with that of blank samples, supporting the preliminary hypothesis that PAHs migrate to different packaging materials, reducing their content in the food contained therein. Furthermore, the use of polytetrafluoroethylene cylindrical packaging for rapeseed oils reduced total PAHs levels from 955.1 to 315.1 μg/kg [66,160].

## 9. Current Knowledge, Future Trends and Recommendations

Several previous studies have addressed the presence of PAHs in foods. However, to the best of our knowledge, the last review article on the subject published in 2019 focused on research trends on PAHs in food until 2017 [123]. Considering the importance and fast-moving pace of this field, constant updates are needed—which we have provided herein. Furthermore, new legislation changes [37] require revisiting and updating the previous knowledge of the presence of PAHs in foods, especially regarding their impact on traditional (e.g., smoked meat and smoked meat products, and smoked fish and smoked fishery products) and emerging (e.g., plant-based powders for beverage preparation) products from the food industry.

This study identified important gaps in the literature and provided perspectives for the design of future studies on PAHs. We highlight the following important aspects and recommendations: (1) global strategies for food production and processing—at both domestic and industrial levels—are necessary to ensure that food products are prepared safely concerning PAHs formation; (2) critical control points in the food production chain must be identified to reduce the formation of PAHs in raw materials and ready-to-eat foods; (3) the HACCP provides a more accurate assessment of the potential risks related to the presence of PAHs in foods, and the application of codes of practice reduces PAHs contamination, especially during smoking and direct drying processes. Best practices put forth by the Codex Alimentarius and European Guides should be used as examples; (4) regarding domestic food preparation, future studies should focus on identifying adequate cooking methods to reduce PAHs formation as well as ingredients (e.g., spices and herbs) and pretreatments (e.g., marinades) to prevent their formation; (5) studies evaluating the seasonal influence on PAHs levels in fresh foods, such as produce and meats, are necessary; (6) considering the hypothesis that PAHs may migrate to the packaging in contact with the food, studies investigating the influence of different packaging materials on PAHs levels should be conducted; (7) technological advancements in analytical methods, especially in the extraction and purification stages, must occur to develop faster, cheaper, and more environmentally friendly strategies to detect PAHs without sacrificing precision, accuracy, and sensitivity; (8) the presence of PAHs in foods must be continually monitored and controlled, including risk assessments related to the consumption of contaminated foods and calculations to determine safe intake parameters; (9) future studies should be conducted to understand how PAHs modify the intestinal microbiota and vice-versa; and (10) public policies and regulations must be reviewed or updated to establish safe limits for PAHs in foods. Several countries have insufficient or no legislation to enforce acceptable levels of PAHs in foods. Therefore, reviews comprising recent data on levels of PAH contamination in foods and risk exposure are essential to aid regulatory agencies in updating or establishing these regulations.

## 10. Conclusions

Several studies and governmental organizations recognize the carcinogenic potential of PAHs. However, the most appropriate cooking methods and strategies to minimize their formation and contamination in food remain unclear. Meanwhile, advances in analytical methods are expected to focus on the development of faster, cheaper, and environmentally friendly techniques. Furthermore, improvements in clean-up and identification methods are necessary to lower the limits of quantification of these complex compounds.

Future studies should focus on minimizing the formation of PAHs during food processing as well as on providing safety strategies for domestic and industrial food preparation and manufacturing. The new European regulation [37] will strongly influence the industrial production of traditional (e.g., smoked meat and its products, and smoked fish and its products) and emerging (e.g., plant-based powders for beverage preparation) products. Finally, public policies and regulations must be reinforced or revisited to establish safe limits of PAHs exposure, including the creation and/or improvement of appropriate legislation.

## Figures and Tables

**Table 1:** Chemical and physical properties of polycyclic aromatic hydrocarbons present in food.

Compound Name	Abbreviation	Chemical Formula	Molecular Weight (g mol^−1^)	Melting Point (°C)	Boiling Point (°C)
5-Methylchrysene	5MC	C_19_H_14_	242.3	118	458
Acenaphthene	ACE	C_12_H_10_	154.2	203	279
Acenaphthylene	ACY	C_12_H_8_	152.2	92–93	265–275
Anthracene	ANT	C_14_H_10_	178.2	218	340–342
Benz[a]anthracene	BaA	C_18_H_12_	228.3	158	438
Benzo[a]pyrene	BaP	C_20_H_12_	252.3	179	495
Benzo[b]fluoranthene	BbF	C_20_H_12_	252.3	168	481
Benzo[c]fluorene	BcF	C_17_H_12_	216.3	125–127	398
Benzo[e]pyrene	BeP	C_20_H_12_	252.3	177	492
Benzo[ghi]perylene	BPe	C_22_H_12_	276.3	273	550
Benzo[j]fluoranthene	BjF	C_20_H_12_	252.3	165–166	480
Benzo[k]fluoranthene	BkF	C_20_H_12_	252.3	216	480
Chrysene	Chr	C_18_H_12_	228.3	254	448
Cyclopenta[cd]pyrene	CPP	C_18_H_10_	226.3	170	439
Dibenzo[a,e]pyrene	DeP	C_24_H_14_	302.4	233–244	592
Dibenz[a,h]anthracene	DBA	C_22_H_14_	278.3	262	535
Dibenzo[a,i]pyrene	DiP	C_24_H_14_	302.4	281–284	594
Dibenzo[a,l]pyrene	DlP	C_24_H_14_	302.4	162	595
Flourene	FLR	C_13_H_10_	166.2	116–117	295
Fluoranthene	FLT	C_16_H_10_	202.3	111	375
Indeno[1,2,3-cd]pyrene	IcP	C_22_H_12_	276.3	164	530
Naphthalene	NAP	C_10_H_8_	128.2	80	218
Phenanthrene	PHN	C_14_H_10_	178.2	100	340
Pyrene	PYR	C_16_H_10_	202.3	156	393–404

Adapted from [15].

**Table 2:** International Agency for Research on Cancer carcinogenicity classification of 16 polycyclic aromatic compounds present in food.

Group	Compound	Evidence
1	Benzo[a]pyrene	The agent (mixture) is carcinogenic to humans
2A	Cyclopenta[cd]pyrene	The agent (mixture) is probably carcinogenic to humans
	Dibenzo[a,l]pyrene	
	Dibenz[a,h]anthracene	
2B	Benzo[b]fluoranthene	The agent (mixture) is possibly carcinogenic to humans
	Benzo[k]fluoranthene	
	Benzo[j]fluoranthene	
	Benz[a]anthracene	
	Chrysene	
	5-Methylchrysene	
	Dibenzo[a,i]pyrene	
	Dibenzo[a,h]pyrene	
	Indeno[1,2,3-cd]pyrene	
3	Benzo[ghi]perylene	The agent (mixture or exposure circumstance) is not classifiable as to its carcinogenicity to humans
	Dibenzo[a,e]pyrene	
	Benzo[c]fluorene	

Adapted from [12].

**Table 3:** Types of food processing.

Type of Heat	Cooking Procedure	Characteristics
Dry	Baking	Indirect heat
Moist	Baking	Indirect heat, conducted by water
Moist	Bain-marie	Indirect baking with heat transfer control
Moist	Deep-frying	Indirect heat, conducted by oil
Dry	Gas grilling	Direct heat, controlled temperature
Dry	Charcoal roasting	Direct heat and smoke, partially controlled temperature
Dry	Smoking	Indirect heat and direct smoke
Dry	Microwave cooking	Indirect heat, heating by dissipation of water molecules
Moist	Stewing	Indirect heat, partially conducted by oil

Adapted from Vintilă [48].

**Table 4:** Summary of PAHs concentrations in various food products.

Product	Processing Method	BaP µg/kg	PAH4 ^a^ µg/kg	Total Sum ^b^ µg/kg	Reference
Milk, Dairy and Baby Foods					
Baby foods (*n* = 40)	None	0.19–0.55	1.02–3.12	11.82–52.25 (14)	[74]
Milk powder (*n* = 44)	None	0.13	2.36	169.99 (16)	[75]
Milk powder formula (*n* = 5)	None	≤LOD	0.24	1.23 (15)	[76]
Smoked mozzarella cheese (*n* = 57)	None	0.09	0.84	1.24 (8)	[77]
Pasteurized milk, UHT milk (*n* = 29)	None	≤LOD–0.10	≤LOD–0.10	5.86–26.60 (16)	[78]
Milk powder (*n* = 31)	None	0.04	0.02–10.16	11.8–78.40 (15)	[79]
Raw milk, Pasteurized milk, UHT milk (*n* = 36)	None	0.25–0.27	1.21–1.59	5.43–7.75 (16)	[80]
Vegetables and Fruits					
Fruits (*n* = 16)	None	≤LOD	≤LOD–0.59	0.19–1.08 (8)	[15]
Vegetables (*n* = 26)	None	≤LOD–0.35	≤LOD–3.28	ND–3.81 (8)	[15]
Vegetables (*n* = 40)	The samples were prepared according to each type of vegetable	≤LOD–4.35	≤LOD–2.00	14.20–413.20 (16)	[81]
Vegetables (*n* = 45)	None	≤LOD–4.40	1.40–19.60	60.50–312.00 (16)	[82]
Vegetables (*n* = 355)	None	0.01–0.14	-	0.20–0.85 (8)	[83]
Meat and Fish					
Chicken wings (*n* = 18)	Grilling (temperature) 270 °C	3.27	8.60	12.83 (8)	[84]
	240 °C	2.51	7.45	11.23 (8)	
	210 °C	1.38	6.59	10.10 (8)	
Fish products (*n* = 18)	Fresh (*n* = 10)	<LOQ	<0.33	<0.33(4)	[85]
	Marinated (*n* = 4)				
	Smoked (*n* = 4)				
Shellfish (*n* = 63)	Fresh	<LOQ–3.43	<0.27–7.92	<0.27–7.92 (4)	
Meat products (*n* = 99)	Fermented sausages (*n* = 21)	<LOQ–5.40	<0.40–7.10	<0.40–7.10 (4)	
	Semi-dry sausages (*n* = 25)	<LOQ–1.05	<0.40–2.53	<0.40–2.53 (4)	
	Pasteurized sausages (*n* = 5)	<LOQ–0.57	0.46–3.31	0.46–3.31 (4)	
	Dry cured meat (*n* = 13)	<LOQ–1.90	0.46–12.66	0.46–12.66 (4)	
	Semi-dry cured meat (*n* = 24)	<LOQ–2.48	<0.40–9.44	<0.40–9.44 (4)	
	Dry bacon (*n* = 8)	<LOQ	<0.40–9.45	<0.40–9.45 (4)	
Meat products (*n* = 35)	None	0.19–1.73	0.59–7.55	0.59–11.05 (8)	[15]
Cocoa and Chocolate					
Cocoa beans (*n* = 8)	Fermented and sun dried	<LOQ–9.06	<LOQ–49.96	<LOQ–63.30 (8)	[86]
Cocoa beans (*n* = 4) and crushed cocoa beans (*n* = 4)	Fermented and dried	≤LOD	≤LOD–0.01	0.38–0.76 (11)	[87]
	135 °C (40 min)				
	135 °C (50 min)				
	150 °C (25 min)				
	150 °C (35 min)				
	135 °C (7 min)				
	135 °C (10 min)				
	150 °C (5 min)				
	150 °C (7 min)				
Milk chocolate (*n* = 7)	None	≤LOD	≤LOD–36.2	48.70–147.00 (13)	[88]
Dark chocolate (*n* = 7)			≤LOD–38.5	87.80–149.00 (13)	
White chocolate (*n* = 6)			≤LOD–17.1	44.20–122.00 (13)	
Raw cocoa beans (*n* = 12)	Unshelled, fermented, and dried	≤LOD–9.98	0.88–44.28	0.88–44.28 (4)	[89]
Cocoa mass (*n* = 22)	Finely ground cocoa nibs	≤LOD–0.76	2.59–9.29	2.59–9.29 (4)	
Cocoa butter (*n* = 67)	Pressed cocoa mass	≤LOD–7.86	2.09–92.53	2.09–92.53 (4)	
Milk chocolate (*n* = 27)	None	0.70	10.11	10.11 (4)	
Dark chocolate (*n* = 69)	None	0.57	5.88	5.88 (4)	
Cocoa powder (*n* = 12)	Finely ground cocoa press cake	1.07	8.86	8.86 (4)	
Cocoa drink powder (*n* = 9)	Finely ground cocoa press cake	1.00	9.48	9.48 (4)	
Chocolate candy (*n* = 25)	None	≤LOD–12.76	≤LOD–17.11	2.70–235.16 (16)	[90]
Cocoa beans (*n* = 8)	Drying and sun drying (65 °C. 70 °C, and 80 °C)	0.15–0.42	-	0.15–0.42 (1)	[91]
Chocolate (*n* = 40)	None	0.07–0.63	0.69–4.45	1.33–6.85 (14)	[92]
Bread and Cereal					
Bread samples (*n* = 10)	Commercial samples	<LOQ–0.20	0.11–0.22	0.11–0.22 (4)	[93]
Breakfast cereals (*n* = 10)	Commercial samples	<LOQ–0.30	0.23–0.87	0.23–0.87 (4)	
Baguette (*n* = 52)	Baked (industrial)	-	0.48–12.55	20.78–228.98 (13)	[94]
Lavash (*n* = 62)	Baked for 80 s at 332 °C	-	0.48–4.94	9.46–152.07 (13)	
Taftoon (*n* = 55)	Baked for 2.5 min at 315 °C	-	0.48–20.66	18.19–169.26 (13)	
Pasta	Commercial samples	≤LOD	≤LOD–920	168–1980 (16)	[95]
Cereal		≤LOD–4200	≤LOD–7500	3500–22,300 (16)	
Flour		≤LOD	≤LOD	803–2486 (16)	
Rice		≤LOD	≤LOD	180–10,540 (16)	
Bread		≤LOD	≤LOD–480	1290–4800 (16)	
Toast	Toasted on an electric home toaster at 600 W for 2 min	≤LOD–980	240–3450	13,000–17,820 (16)	

Values are presented as mean or range, as appropriate. ^a^ PAH4: benz[a]anthracene, benzo[a]pyrene, benzo[b]fluoranthene, and chrysene. ^b^ The number in parentheses indicates the number of compounds included in the sum. LOD: limit of detection, LOQ: limit of quantification.

**Table 5:** Recent techniques for the determination of polycyclic aromatic hydrocarbons in food products.

Food Product	Extraction	Clean-Up Step	Quantification	Reference
Smoked fish and meat	Saponification (3.5 M methanolic potassium hydroxide solution at 70 °C for 2 h) and cyclohexane	Silica SPE cartridge	GC–QMS	[63]
Dry/fermented sausage	Saponification (potassium hydroxide, methanol and water for 3 h under reflux) and extraction with *n*-hexane	Florisil SPE cartridge	UHPLC-UV/VIS/FLD	[60]
Beef meat	Saponification (sodium hydroxide and diatomaceous earth)	*Extrelut* column connected to a PRS SPE column and silica SPE cartridge	HPLC-FLD	[129]
Beef stripe, pork, and chicken fillet	Saponification (2 M methanolic potassium hydroxide solution) and extraction with *n*-hexane	SPE in neutral-Si/basic-Si/acidic-Si/neutral-Si frits	GC–FID	[104]
Dry fermented sausage	Acetonitrile, magnesium sulfate, and sodium chloride	QuEChERS (C18 + PSA)	GC-QMS	[61]
Olive and refined pomace olive oils	Acetone/acetonitrile in an alumina-N SPE cartridge	Amine SPE cartridge	HPLC-FLD	[130]
Salmon, shrimps, mussels, cutlet, bacon, curry spice, wheat flour, infant formula, cereal-based baby foods with fruits, and baby foods with vegetables	QuEChERS (Acetonitrile, and salts combinations)	QuEChERS (C18 + PSA), EMR-Lipid	GC- QqQMS	[131]
Milk and meat/fish-based baby food	Saponification (1 M ethanolic potassium hydroxide solution at 80 °C for 3 h)	Washed the cyclohex-ane phase with meth-anol:water	HPLC-FLD	[74]
Chicken breast, smoked ham, ham, roasted bacon, crispy bacon, pork sausage, Swedish meatballs, honey roast salmon, and sweet chili salmon	Saponification (sodium hydroxide and diatomaceous earth)	Silica SPE cartridge	HPLC-DAD/FLD	[106]
Spices and meat	Homogenized with 1 M sodium hydroxide solution and *Extrelut* and loaded in an *Extrelut* 20 column, followed by PRS cartridges with dichloromethane containing 5% toluene	Silica SPE cartridge	HPLC-FLD	[106]
Fruit, vegetables, and meats and their products	Saponification (ethanolic potassium hydroxide solution 80 °C for 3 h) and extraction with *n*-hexane–ethanol solution	Silica SPE cartridge	GC-QMS	[15]
Fish and malt	Acetonitrile and ceramic stone	QuEChERS (Zirconium dispersive SPE)	GC-QTOF MS	[132]
Fish, mollusk, and shrimp	ELL with ethyl acetate and aqueous solution containing magnesium sulfate and sodium chloride	Silica SPE cartridge	GC-MS	[133]
Dark chocolate	Dichloromethane and *n*-hexane	Gel permeation chromatography		[134]
Animal-based smoked foods	QuEChERS (Acetonitrile, water, and salts combinations)	QuEChERS (C18 + PSA), EMR-Lipid, DLLME	GC-Iontrap MS	[135]
Extruded wheat flour, smoked fish, dry infant formula, sausage meat, freeze-dried mussels, edible oil, and wheat flour	*n*-hexane or cyclohexane	SEC and SPE	GC-MS	[136]
Edible olive oil, fresh mussels, smoked fish, smoked meat products, processed cereal-based foods for young children, infant formula, chocolate, and food supplements (isoflavones)	Dichloromethane	SEC	HPLC-FLD	[137]

DLLME, dispersive liquid-liquid micro-extraction; ELL, extraction liquid-liquid; EMR, enhanced matrix removal; FID, flame ionization detector; FLD, fluorescence detector; GC, gas chromatograph; HPLC, high performance liquid chromatography; MS, single quadrupole, triple quadrupole, time-of-flight, or ion trap mass spectrometer; PRS, propyl sulphonic acid; PSA, primary secondary amine; SPE, solid-phase extraction; QMS, single quadrupole mass spectrometer, QqQMS, triple quadrupole mass spectrometer; QTOF MS, time-of-flight mass spectrometer; QuEChERS, quick, easy, cheap, effective, rugged and safe method (solvent, salts combinations, and dispersive solid phase extraction sorbents); SEC, size-exclusion chromatography; UHPLC, ultrahigh performance liquid chromatography; UV, ultraviolet absorption detector; VIS, visible absorption detector.
